# Long-term effects of mild traumatic brain injury on cognitive performance

**DOI:** 10.3389/fnhum.2013.00030

**Published:** 2013-02-12

**Authors:** Philip J. A. Dean, Annette Sterr

**Affiliations:** Department of Psychology, University of SurreyGuildford, UK

**Keywords:** head injury, minor, post-concussion syndrome, cognition, neuropsychological tests

## Abstract

Although a proportion of individuals report chronic cognitive difficulties after mild traumatic brain injury (mTBI), results from behavioral testing have been inconsistent. In fact, the variability inherent to the mTBI population may be masking subtle cognitive deficits. We hypothesized that this variability could be reduced by accounting for post-concussion syndrome (PCS) in the sample. Thirty-six participants with mTBI (>1 year post-injury) and 36 non-head injured controls performed information processing speed (Paced Visual Serial Addition Task, PVSAT) and working memory (n-Back) tasks. Both groups were split by PCS diagnosis (4 groups, all *n* = 18), with categorization of controls based on symptom report. Participants with mTBI and persistent PCS had significantly greater error rates on both the n-Back and PVSAT, at every difficulty level except 0-Back (used as a test of performance validity). There was no difference between any of the other groups. Therefore, a cognitive deficit can be observed in mTBI participants, even 1 year after injury. Correlations between cognitive performance and symptoms were only observed for mTBI participants, with worse performance correlating with lower sleep quality, in addition to a medium effect size association (falling short of statistical significance) with higher PCS symptoms, post-traumatic stress disorder (PTSD), and anxiety. These results suggest that the reduction in cognitive performance is not due to greater symptom report itself, but is associated to some extent with the initial injury. Furthermore, the results validate the utility of our participant grouping, and demonstrate its potential to reduce the variability observed in previous studies.

## Introduction

A number of studies report that mild traumatic brain injury (mTBI) participants have reduced cognitive performance, even in the long-term (>3 months) after injury, on tasks that assess attention (Mangels et al., 2002; Chan, 2005; Kumar et al., 2005; Sterr et al., 2006; Catale et al., 2009; Maruta et al., 2010), memory (Vanderploeg et al., 2005; Chen et al., 2007; Catale et al., 2009), executive function (O'Jile et al., 2006; Sterr et al., 2006; Erez et al., 2009; Pontifex et al., 2009), and information processing (O'Jile et al., 2006; Lachapelle et al., 2008; Johansson et al., 2009; Kinnunen et al., 2011). However, there is also research that observes no deficit in cognitive performance in mTBI patients in the long-term (Chen et al., 2004; Perlstein et al., 2004; Solbakk et al., 2005; Broglio et al., 2009; Tellier et al., 2009) or within the 3 months following injury (McAllister et al., 2001; Chen et al., 2004; Jantzen et al., 2004; Lange et al., 2009; Tellier et al., 2009; Slobounov et al., 2010). Indeed, even in those investigations that do detect a deficit, there seems to be little consistency in which cognitive performance is impaired (Tellier et al., 2009). Inconsistency and variability between previous studies on long-term cognitive performance after mTBI is likely to be due to a combination of the variety of tasks used and the distinct samples tested.

A variety of different aspects of cognitive performance have been investigated in the long-term after mTBI, using a number of different tasks. More importantly, tasks assessing the same cognitive function have varied in their difficulty, possibly leading to the inconsistent results. A challenging cognitive task may be required to observe the subtle long-term alterations in participants with mTBI (Segalowitz et al., 2001; Chen et al., 2004). Of particular utility in this regard are tasks that can be parametrically increased in difficulty (Braver et al., 1997; Pare et al., 2009), enabling an investigation of the effects of enhancing cognitive load.

Two tasks that can be parameterized in this way are the n-Back (assessing working memory) and Paced Visual Serial Addition Task [PVSAT, assessing information speed (Fos et al., 2000)]. Both of these tasks have been previously used in mTBI research [n-Back: (McAllister et al., 1999, 2001; Wang et al., 2006; Catale et al., 2009); PVSAT: (Cicerone and Azulay, 2002; Vanderploeg et al., 2005; O'Jile et al., 2006; Wang et al., 2006; Mayer et al., 2009; Brenner et al., 2010b)], with the paced auditory serial addition task specifically created to investigate cognitive difficulties after TBI. However, few of the previous studies have used a range of difficulties within PVSAT to assess cognition.

In addition, sampling of a mTBI population is challenging, as there is inherent heterogeneity between individuals (Shum et al., 2011) with differing severity of injury and subsequent outcome. One way of reducing variability is to split the mTBI sample by post-concussion syndrome (PCS) diagnosis (WHO, 1992), as has been argued previously (Hartlage et al., 2001; Cicerone and Azulay, 2002; Wang et al., 2006). Studies that have split their mTBI sample by PCS diagnosis have been relatively more consistent in their findings of cognitive deficit (Cicerone and Azulay, 2002; Kumar et al., 2005; Sterr et al., 2006; Wang et al., 2006; Chen et al., 2007; Ptito et al., 2007; Johansson et al., 2009).

PCS is the term for the range of cognitive, somatic, and affective symptoms usually reported by participants after a mTBI (Ryan and Warden, 2003). Symptoms typically resolve within 3 months (Korinthenberg et al., 2004; Lundin et al., 2006; Lannsjo et al., 2009; Sigurdardottir et al., 2009; Yang et al., 2009; Sroufe et al., 2010), but in some individuals these symptoms persist (Killam et al., 2005; Sterr et al., 2006; Stulemeijer et al., 2007; Hessen et al., 2008), and can be present years after injury (Vanderploeg et al., 2005). However, PCS symptom report is influenced by depression (Suhr and Gunstad, 2002; Iverson, 2006), chronic pain (Radanov et al., 1992), post-traumatic stress (Hoge et al., 2008; Nelson et al., 2009; Kennedy et al., 2010), anxiety (Moore et al., 2006), fatigue (Johansson et al., 2009), and involvement in litigation (Greiffenstein and Baker, 2001; Lees-Haley et al., 2001). Post-concussion-like symptoms have also been reported in healthy participants at levels that would result in PCS diagnosis in a head injured population (Chan, 2001; Iverson and Lange, 2003; Wang et al., 2006; Fear et al., 2009). Furthermore, symptoms such as memory and concentration problems have been shown to emerge during the early recovery phase rather than forming the initial symptom complex (Dikmen et al., 2010; Meares et al., 2011).

Consequently, there is some debate whether persistent PCS (>3 months) is due to biological factors from neural damage or a psychological response to the mTBI (Mittenberg et al., 1992; Bailey et al., 2006; Mulhern and McMillan, 2006). It has been shown that subjective symptom report does not relate to objective symptoms (Nolin et al., 2006; Spencer et al., 2010). This has led some to suggest that PCS is not specific to mTBI (Sroufe et al., 2010), a finding we recently confirmed on a larger sample of 350 participants (Dean et al., 2012). However, the use of adequate control populations can help alleviate some of the problems associated with the non-specificity of PCS. Previous studies have used specific clinical populations such as those with post-traumatic stress disorder (PTSD), chronic pain, and patients with equivalent injuries to the body, sparing the head (Bell et al., 1999; Vanderploeg et al., 2009; Meares et al., 2011). It is also possible to control for post-concussion-like symptoms in healthy participants by splitting this group by PCS in a similar way to those with mTBI. Healthy control participants with levels of symptoms that would result in PCS diagnosis can then be compared to those mTBI participants with PCS. Cognitive differences between these two groups may then be attributed to the report of PCS after mTBI, and not the symptoms alone. Furthermore, if PCS is induced to some extent by damage at the time of injury, then it can be assumed that those mTBI participants with greater symptoms will perform worse on cognitive tasks, whereas there will be no correlation between performance and symptoms in control participants.

Based on the considerations above, the present study investigates working memory and information processing speed in participants a year or more post-mTBI compared to a non-head injured control population. Both populations were assessed for PCS symptoms, and split into those with and without on-going PCS to form four participants groups: mTBI + PCS, mTBI − PCS, Control + PCS, and Control − PCS. Control participants are labeled as having PCS when they meet all the DSM-IV criteria (APA, 1994), with the exception of previous head injury.

These four groups were used to test the hypothesis that only participants who report persistent PCS after mTBI will show a cognitive deficit. In contrast, head-injured individuals who report no on-going PCS symptoms, and those without prior head injury (regardless of extent of post-concussion symptoms) are likely to have no evidence of cognitive dysfunction. Furthermore, the cognitive deficit in mTBI participants with PCS will become more apparent as the difficulty of the task is parametrically increased.

## Materials and methods

### Participants

#### Recruitment

The study specifically aimed to recruit persons who had not sought medical attention following their mTBI. A large number of those who sustain a mTBI are unreported in traditional hospital and emergency department-based research (Segalowitz and Lawson, 1995; NCIPC, 2003; Bazarian et al., 2005). Consequently, participants were recruited from a database generated by a previous study (Dean et al., 2012) which used an online survey aimed at the general public. This survey was open to both those with and without head injury, and recorded demographic information, comprehensive details about any prior head injury (in order to determine whether any injury met the diagnosis criteria for mTBI), and questionnaires on PCS and co-variables as detailed below. Those reporting any form of head injury in the survey were subsequently screened for mTBI according to ICD-10 criteria. The study protocol was given a favorable opinion by the University of Surrey Ethics Committee. Written informed consent was obtained prior to participation.

#### Diagnosis

We determined mTBI using ICD-10 criteria (Holm et al., 2005). According to these criteria, participants must report one or more of the following: dizziness or confusion; loss of consciousness ≤30 min; post-traumatic amnesia <24 h. A case history was taken which included a description of the injury, the date of injury, any other head injuries as well as general health and lifestyle information. Only participants at least a year post-mTBI, with no report of litigation, major invasive head injury, chronic pain, or other neurological conditions were contacted to take part in the study. Control participants were selected as those who did not report any prior head injury.

We diagnosed PCS using the modified DSM-IV criteria specified by Mittenberg and Strauman (2000), which requires report of three or more of the following symptoms subsequent to head trauma: (1) headache, (2) vertigo or dizziness, (3) irritability or aggression on little or no provocation, (4) anxiety, depression, or affective instability, (5) becoming fatigued easily, (6) disordered sleep, (7) changes in personality, and (8) apathy or lack of spontaneity. The extent of PCS was measured using the Rivermead Post-Concussion symptoms Questionnaire [RPQ; (King et al., 1995)] and Rivermead Post-Concussion symptoms Questionnaire for Controls [RPQ-C; (Sterr et al., 2006; Dean et al., 2012)]. PCS diagnosis was achieved in the same way for control participants as mTBI participants, with the exception that controls had no “history of head trauma.” The majority of control participants did not attribute their symptoms to any specific cause, with a few (*n* = 5) attributing them to generalized stress or anxiety.

#### Study groups

Once diagnosed, selected participants were then asked to take part in computer-based tasks of memory and mental agility. Four groups with 18 participants each were included in this study (for demographics, see Table 1). The groups were:

- *mTBI + PCS*: Participants who suffered a mTBI and have persistent PCS
- *mTBI − PCS*: Participants with mTBI but no current PCS (this does not preclude them having had acute PCS symptoms that have recovered)
- *Control + PCS*: Participants with PCS, but no history of brain injury
- *Control − PCS*: Participants with no history of brain injury and no PCS

**Table 1:** **Demographic and questionnaire data**.

	**mTBI**		**Control**		**Group difference**
	**PCS**	**No PCS**	**PCS**	**No PCS**	
Age	26.7 ± 2.1	26.6 ± 1.8	24.4 ± 1.5	26.7 ± 2.5	–
Gender (F/M)	12/6	10/8	10/8	10/8	–
IQ (NART)	112.1 ± 1.1	116.1 ± 1.0	115 ± 1.4	116.0 ± 1.0	–
RPQ	24.6 ± 1.8	8.8 ± 1.5	27.7 ± 2.5	2.9 ± 0.7	*p* < 0.001
CFQ	55.8 ± 4.4	39.0 ± 3.2	45.3 ± 3.5	25.6 ± 2.9	*p* < 0.001
HADS: Anxiety	7.8 ± 0.7	5.6 ± 1.1	10.1 ± 1.1	5.6 ± 0.7	*p* = 0.002
HADS: Depression	4.1 ± 0.7	2.1 ± 0.5	5.3 ± 0.8	2.0 ± 0.6	*p* = 0.001
ESS	8.1 ± 1.3	6.9 ± 0.9	8.6 ± 1.1	5.6 ± 0.6	–
KSS: Pre	3.9 ± 0.3	4.1 ± 0.4	4.6 ± 0.4	3.2 ± 0.2	–
KSS: Post	5.1 ± 0.4	5.4 ± 0.4	5.6 ± 0.4	4.8 ± 0.4	–
KSS: Post-Pre	1.2 ± 0.4^*^	1.3 ± 0.5^*^	1.0 ± 0.3^*^	1.6 ± 0.5^*^	–
PSQI	8.6 ± 0.8	4.5 ± 0.6	6.6 ± 0.7	5.5 ± 0.6	*p* = 0.001
IES-R	23.9 ± 5.7	6.4 ± 2.6	–	–	*p* = 0.009

Note: All groups: n = 18, except NART (mTBI + PCS: n = 14; mTBI − PCS: n = 17; Control + PCS: n = 13; Control − PCS: n = 16) and PSQI (mTBI + PCS: n = 12; mTBI − PCS: n = 16; Control + PCS: n = 14; Control − PCS: n = 13). All figures except Gender expressed as mean ± SEM. Shaded gray boxes indicate groups generating the significant difference as revealed by Bonferroni-adjusted pairwise comparisons.

* KSS Post-significantly greater than KSS Pre in all groups (p < 0.05). NART, National Adult Reading Test; RPQ, Rivermead Post-Concussion Symptoms Questionnaire; CFQ, Cognitive Failures Questionnaire; HADS, Hospital Anxiety and Depression Scale; ESS, Epworth Sleepiness Scale; KSS, Karolinska Sleepiness Scale; PSQI, Pittsburgh Sleepiness Index; IES-R, Impact of Event Scale-Revised.

### Questionnaires

In addition to the RPQ, questionnaires that assessed common co-variables of PCS were included in the survey (Dean et al., 2012): everyday cognitive failures [Cognitive Failures Questionnaire, CFQ; (Broadbent et al., 1982)], daytime sleepiness [Epworth Sleepiness Scale, ESS; (Johns, 1991)], PTSD [Impact of Event Scale – Revised, IES-R; (Weiss, 2007)], anxiety, and depression [Hospital Anxiety and Depression Scale, HADS; (Bjelland et al., 2002)]. A measure of sleep propensity [Karolinska Sleepiness Scale, KSS; (Gillberg et al., 1994)] was taken before (KSS Pre) and after (KSS Post) the behavioral task. Overall sleep quality [Pittsburgh Sleep Quality Index, PSQI; (Buysse et al., 1989)] and IQ [National Adult Reading Test, NART; (Nelson, 1982)] were assessed either on the day of cognitive testing or on a subsequent day due to time constraints. Therefore, not all participants could complete these two assessments: PSQI (mTBI + PCS: *n* = 12; mTBI − PCS: *n* = 16; Control + PCS: *n* = 14; Control − PCS: *n* = 13) and NART (mTBI + PCS: *n* = 14; mTBI − PCS: *n* = 17; Control + PCS: *n* = 13; Control − PCS: *n* = 16).

### Cognitive tasks

Participants were presented with two behavioral tasks: the n-Back and the PVSAT. Both tasks looked identical: single digit numbers between 1 and 9 inclusive were presented on the screen one at a time, with 60 of these stimuli (including 20 randomly ordered target stimuli) per block. There was a total of 12 blocks for each task, with 3 randomly ordered repetitions of the 4 levels of difficulty. The order of presentation (n-Back/PVSAT) was counterbalanced across participants. The keys M and C on a standard keyboard were counterbalanced as target and non-target response buttons across the participants.

#### n-Back

There were four conditions: 0-Back, 1-Back, 2-Back, and 3-Back. The numbers were presented every 3 s. Participants were asked to press the target button when the number on screen matched the number observed one previous (1-Back), two previous (2-Back), or three previous (3-Back). For every other number that did not match, participants were asked to press the non-target button. In the fourth condition (0-Back) a random number between 1 and 9 was designated as a target at the beginning of the block. Performance on the 0-Back condition should be near ceiling for all participant groups, and was therefore used as a test of performance validity.

#### PVSAT

There were four conditions: 2.5 s PVSAT, 2 s PVSAT, 1.5 s PVSAT, and 1 s PVSAT. The inter-stimulus interval (ISI) was 2.5 s, 2 s, 1.5 s, or 1 s. Each of the four ISI's was presented with each of the three target numbers. Participants were required to add the number on screen to the previously presented number. At the beginning of each block they were given a target number of 9, 10, or 11. If the addition equalled the target number, a “correct” response was required. An “incorrect” response was required for every other addition.

### Data analysis

A series of One-Way ANOVAs were carried out on the questionnaire and demographic data (Table 1), with between-subjects factor of GROUP and *post-hoc* Bonferroni-corrected comparisons. Paired samples *t*-tests were performed for each of the groups to assess the difference between KSS Pre and Post. Gender differences were assessed using a χ^2^ test.

The cognitive tasks were analyzed using two separate mixed-model ANOVAs with factor of DIFFICULTY LEVEL (3-, 2-, 1-, 0-Back or 1, 1.5, 2, 2.5 s) and between-subjects factor of GROUP (mTBI + PCS, mTBI − PCS, Control + PCS, Control − PCS), with *post-hoc* Bonferroni-corrected comparisons as appropriate. Subsequent to this, a series of One-Way ANOVAs were performed for each difficulty level within each task.

In order to investigate the contribution of post-concussion symptoms and its co-variables to cognitive performance after head injury, a series of Spearman's Rho (ρ) correlations were performed. The sample was split into those with mTBI (*n* = 36) and those without (*n* = 36), and average error rates across conditions were calculated as a measure of global performance (n-Back average did not include 0-Back). Only those co-variables which significantly differed between groups were used in the analysis. Correction for multiple comparisons was used, with a modified threshold *p*-value of 0.002.

## Results

### Demographics and questionnaire measures

There was no significant difference between the groups on any of the demographic data (age, gender, IQ). However, a significant effect of GROUP was observed for post-concussion symptoms [RPQ; *F*_(3, 68)_ = 47.8, *p* < 0.001], cognitive failures [CFQ; *F*_(3, 68)_ = 12.7, *p* < 0.001], anxiety [HADS; *F*_(3, 68)_ = 5.7, *p* = 0.002], depression [HADS; *F*_(3, 68)_ = 6.4, *p* = 0.001], nocturnal sleep quality [PSQI; *F*_(3, 51)_ = 6.8, *p* = 0.001], and PTSD [IES-R; *F*_(1, 33)_ = 7.7, *p* = 0.009].

Bonferroni-adjusted pairwise comparisons revealed no difference on any questionnaire measure between mTBI + PCS and Control + PCS participants, suggesting that their subjective symptom report was similar. This was also true for the comparison between mTBI − PCS and Control − PCS participants. The observed group differences were caused by higher symptom report in the groups with high PCS symptoms compared to those with low PCS symptoms (Table 1), as expected.

In detail, higher RPQ and CFQ symptom report was observed in mTBI + PCS and Control + PCS participants compared to mTBI − PCS and Control − PCS (RPQ: all *p* < 0.001; CFQ: all *p* < 0.01), with the exception of the comparison of CFQ score between Control + PCS and mTBI − PCS participants (*p* = 1.0). Anxiety and depression scores were higher only in Control + PCS participants compared to both mTBI − PCS (anxiety: *p* = 0.005; depression; *p* = 0.004) and Control − PCS (anxiety: *p* = 0.005; depression: *p* = 0.003). Nocturnal sleep quality was lower in mTBI + PCS participants compared to both mTBI − PCS (*p* < 0.001) and Control − PCS (*p* = 0.018) participants. Lastly, mTBI + PCS participants reported a greater number of PTSD symptoms compared to mTBI − PCS participants (*p* = 0.009).

Control + PCS participants had borderline abnormal levels of depression, but anxiety within normal bounds. Mean PSQI scores for mTBI + PCS and Control + PCS participants were indicative of poor nocturnal sleep. However, the two groups without PCS had borderline scores, suggesting a generally poor level of nocturnal sleep in the sample. All groups reported greater sleep propensity after the task compared to the beginning [KSS Post-Pre: −mTBI + PCS: *t*_(18)_ = 2.3, *p* = 0.036; mTBI − PCS: *t*_(18)_ = 2.6, *p* = 0.020; Control + PCS: *t*_(18)_ = 3.4, *p* = 0.003; Control − PCS: *t*_(18)_ = 3.6, *p* = 0.002], suggesting that the task was uniformly tiring.

### Cognitive tasks

#### Error rates

A main effect of GROUP was seen for both the n-Back [*F*_(3, 68)_ = 8.3, *p* < 0.001] and PVSAT [*F*_(3, 68)_ = 9.8, *p* < 0.001] tasks, together with an interaction between GROUP and DIFFICULTY LEVEL for the n-Back only [*F*_(7, 150)_ = 3.5, *p* = 0.002; PVSAT: *F*_(7, 149)_ = 0.8, *p* = 0.55]. As expected, there was a main effect of DIFFICULTY LEVEL [n-Back: *F*_(2, 150)_ = 114.3, *p* < 0.001; PVSAT: *F*_(2, 149)_ = 150.2, *p* < 0.001], reflecting greater error rates with each increase in difficulty level (all comparisons: *p* < 0.001, except 2 vs. 2.5: *p* = 0.037).

**Figure 1:**
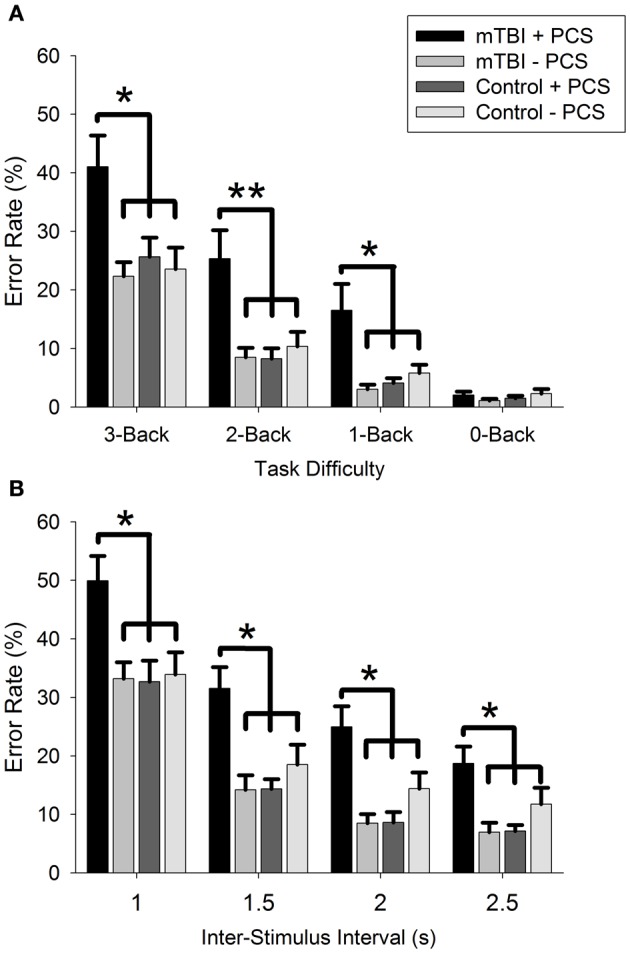
**Error rate for (A): n-Back and (B): PVSAT tasks.** ^*^p < 0.05, ^**^p < 0.01.

Bonferroni-adjusted pairwise comparisons revealed that participants with mTBI and PCS produced significantly greater error rates than all other groups (see Figure 1) for the n-Back [mTBI − PCS: mean difference (MD) = 12.5, *p* < 0.001; Control + PCS: MD = 11.4, *p* = 0.001; Control − PCS: MD = 10.8, *p* = 0.002] and the PVSAT (mTBI − PCS: MD = 15.6, *p* < 0.001; Control + PCS: MD = 5.6, *p* < 0.001; Control − PCS: MD = 11.6, *p* = 0.005). All other comparisons were not statistically significant (all *p* = 1.0).

A further series of ANOVA examined whether these GROUP differences were observed for each DIFFICULTY LEVEL in isolation. These revealed a main effect of GROUP for every condition [3-Back: *F*_(3, 68)_ = 5.2, *p* = 0.003; 2-Back: *F*_(3, 68)_ = 7.5, *p* < 0.001; 1-Back: *F*_(3, 68)_ = 6.6, *p* = 0.001; 1 s: *F*_(3, 68)_ = 5.2, *p* = 0.003; 1.5 s: *F*_(3, 68)_ = 7.9, *p* < 0.001; 2 s: *F*_(3, 68)_ = 9.3, *p* < 0.001; 2.5 s: *F*_(3, 68)_ = 6.1, *p* = 0.001], with the exception of 0-Back [*F*_(3, 68)_ = 0.9, *p* = 0.47]. Again, it was the mTBI + PCS group that produced greater error rates than all other groups for the n-Back (3-Back: all *p* < 0.05; 2-Back: all *p* < 0.005; 1-Back: all *p* < 0.05) and the PVSAT (1 s: all *p* < 0.05; 1.5 s: all *p* < 0.05; 2 s: all *p* < 0.05). However, in the 2.5 s condition, mTBI + PCS produced significantly greater error rates than mTBI − PCS (MD = 11.8, *p* = 0.002) and Control + PCS (MD = 11.6, *p* = 0.003), but not Control − PCS (MD = 11.6, *p* = 0.18).

#### Reaction time

**Figure 2:**
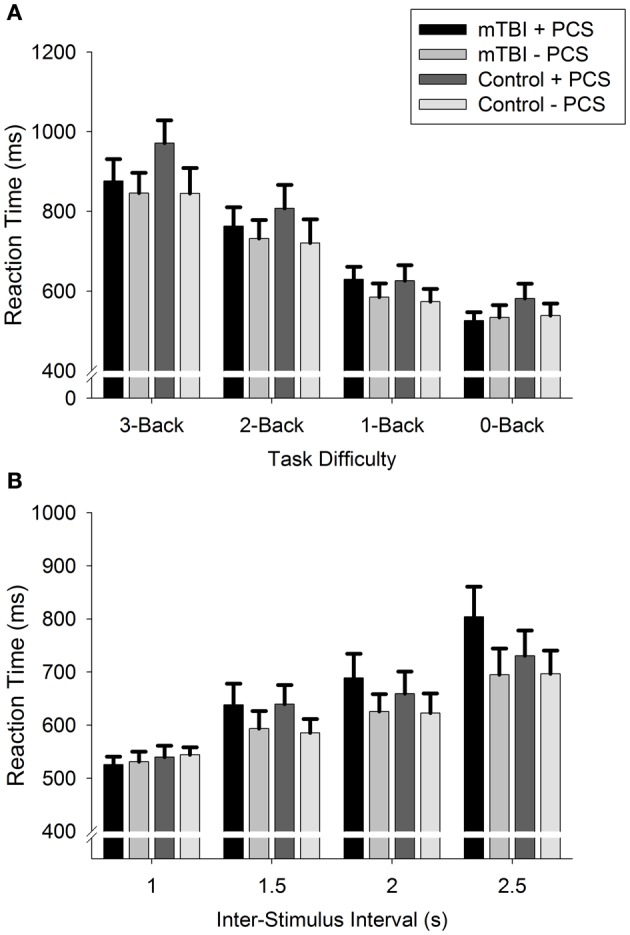
**Reaction Time for (A): n-Back and (B): PVSAT tasks**.

For both tasks, the main effect of GROUP and the GROUP × DIFFICULTY LEVEL interaction were not statistically significant (Figure 2). However, there was a main effect of DIFFICULTY LEVEL [n-Back: *F*_(2, 123)_ = 149.7, *p* < 0.001; PVSAT: *F*_(2, 119)_ = 59.4, *p* < 0.001], with participants responding slower on the n-Back and faster on the PVSAT as task difficulty increased (all comparisons: *p* < 0.001).

### Correlation between subjective symptom report and objective cognitive performance

Correlation between greater PCS symptom report and poorer task performance was not statistically significant (after multiple comparison correction) in mTBI participants for the PVSAT task (Rho = 0.35, *p* = 0.02, Table 2), nor the n-Back task (Rho = 0.43, *p* = 0.004). Although the n-Back association (*p* = 0.004) fell short of the significance threshold (*p* = 0.002), it represents a medium size effect according to Cohen's (Cohen, 1988) interpretation criteria, along with the PVSAT association. No significant correlations with cognitive performance were observed in control participants for any co-variable.

**Table 2:** **Correlations between symptom report and cognitive task performance**.

**Group**	**Error rates**		**RPQ**	**CFQ**	**HADS anxiety**	**HADS depression**	**PSQI**	**IES-R**
mTBI	n-Back	Rho	0.43	0.18	0.27	0.17	0.31	0.45
		*p*	0.004	0.15	0.06	0.16	0.05	0.004
	PVSAT	Rho	0.35	0.25	0.44	0.33	0.62	0.28
		*p*	0.02	0.07	0.004	0.02	<0.001	0.06
Control	n-Back	Rho	0.10	0.28	0.28	0.29	−0.02	-
		*p*	0.29	0.05	0.05	0.04	0.46	-
	PVSAT	Rho	0.03	0.26	−0.01	0.03	0.32	-
		*p*	0.42	0.06	0.47	0.44	0.05	-

Note: All correlations n = 36, except PSQI (mTBI: n = 28, control: n = 27). Error rates refers to average error rates across all conditions for each task (n-Back average does not include 0-Back). Shaded dark gray boxes indicate significant correlations after multiple comparison correction; light gray boxes indicate correlations that approach significance.

RPQ, Rivermead Post-Concussion Symptoms Questionnaire; CFQ, Cognitive Failures Questionnaire; HADS, Hospital Anxiety and Depression Scale; PSQI, Pittsburgh Sleepiness Index; IES-R, Impact of Event Scale-Revised.

However, there was a significant correlation between lower sleep quality (PSQI) and poorer performance on the PVSAT task (Rho = 0.62, *p* < 0.001) for mTBI participants. There were medium size effects for correlations between poor PVSAT performance and higher anxiety (Rho = 0.44, *p* = 0.004) and poor n-Back performance and high post-traumatic stress symptoms (Rho = 0.43, *p* = 0.004), though these associations fell short of the significance threshold.

## Discussion

### Principal findings

This study demonstrated working memory and information processing speed impairments in participants with mTBI and persistent (>1 year post-injury) PCS. Cognitive performance was similar in mTBI participants without PCS and all non-head injured participants. Critically, this is despite the Control + PCS group displaying comparable subjective report of post-concussion symptoms, cognitive failures, depression, anxiety, and sleep quality to the mTBI + PCS participants. This suggests that the cognitive deficit seen in the mTBI + PCS group is not a result of high PCS symptom report per se, nor a result of the co-variables associated with PCS, but is perhaps due to the combination of ongoing PCS symptom report after initial injury. Therefore, PCS symptoms may have a differential cause, with the mechanisms leading to PCS after mTBI distinct from those contributing to the PCS symptoms seen in the general population.

Although there are some studies on cognitive performance after mTBI that have taken PCS into consideration (Chan, 2001; Wang et al., 2006; Ptito et al., 2007), there are none to our knowledge that have controlled for PCS in both mTBI and control participants. The latter allows a tentative dissociation of the effect of PCS symptom report subsequent to mTBI on cognitive performance from the influence of post-concussion-like co-variables observed in non-head injured populations.

### Cognitive tasks

Participants in the mTBI + PCS group were impaired on both the n-Back (working memory) and the PVSAT task (information processing speed). In contrast to our hypothesis, participants in the mTBI + PCS group were impaired on even the least cognitively demanding working memory (1-, 2-, 3- Back; Figure 1A) and information processing speed conditions (2.5, 2, 1.5, 1 s PVSAT, Figure 1B). It was assumed that the cognitive deficit would be relatively subtle, and only become apparent when task difficulty is high.

**Figure 3:**
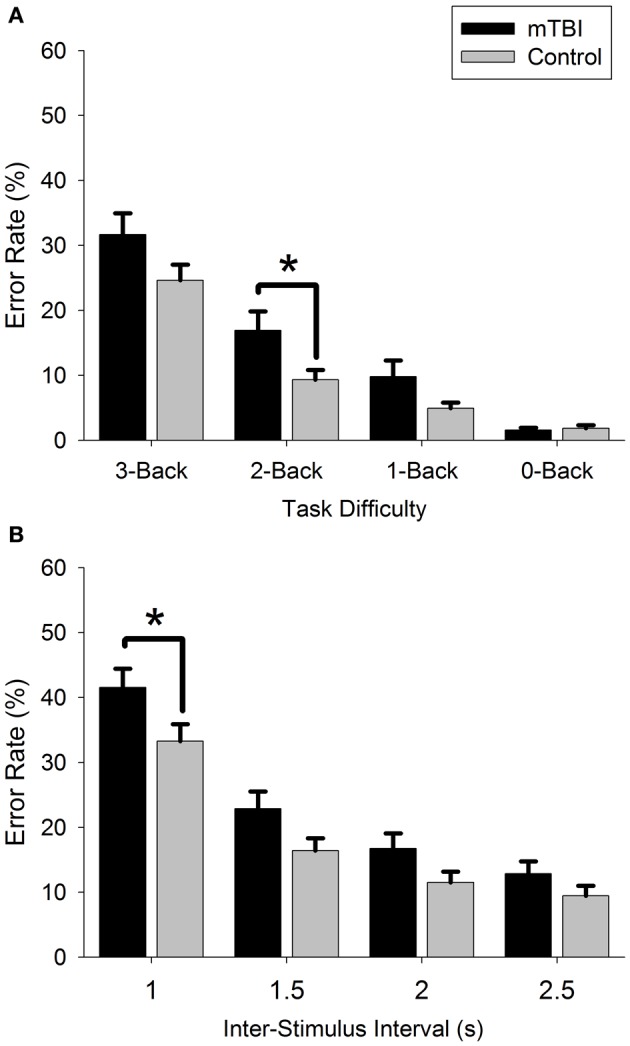
**Comparison of mTBI and control error rate for (A): n-Back and (B): PVSAT tasks.** ^*^p < 0.05.

However, many previous studies have not accounted for PCS diagnosis, potentially masking cognitive impairments in a proportion of participants with mTBI. This certainly seems to be the case if the current dataset is re-analyzed without taking account of PCS (Figure 3; 2 groups: mTBI, *n* = 36; Control, *n* = 36).

Whilst there is still an overall effect of GROUP for both the n-Back [*F*_(1, 70)_ = 4.6, *p* = 0.036] and PVSAT [*F*_(1, 70)_ = 4.7, *p* = 0.034], there is no interaction between GROUP and DIFFICULTY LEVEL, and the GROUP difference is only significant for the 2-Back [*F*_(1, 70)_ = 5.4, *p* = 0.023] and 1 s PVSAT [*F*_(1, 70)_ = 4.5, *p* = 0.037]. Therefore, not taking PCS into account leads to the more subtle results we expected, with only the more difficult levels differentiating between groups. These results suggest that accounting for PCS diagnosis may help reduce the variability inherent to mTBI, and create more consistent results in future research.

An important aspect of the results was that all participant groups performed near ceiling on the 0-Back condition, and there was no significant difference in error rate. This condition was used as an indication of performance validity, and the result suggests this is unlikely to have significantly contributed to the differences observed for working memory and information processing speed. However, the 0-Back is not a standardized measure of effort testing, such as the Test of Memory Malingering (TOMM; Tombaugh, 1996) and Victoria Symptom Validity Test (VSVT; Slick et al., 1997), or even tests with embedded effort sensitive measures such as the Wechsler Adult Intelligence Scale (WAIS; Iverson and Tulsky, 2003) or the Repeatable Battery for the Assessment of Neuropsychological Status (RBANS; Silverberg et al., 2007). As such, it is possible that this test may not be able to detect poor effort in the symptomatic group. However, participants had no overt incentive for poor effort, as they had been screened for any litigation and on-going chronic pain. Previous studies have suggested that participants without overt incentives for poor effort only fail standardized effort tests in a small proportion of cases (Kemp et al., 2008; Pella et al., 2012). This could be due to there being no difference in effort in these groups, due to the difference being so slight that it is not detectable, or due to the standardized tests not being suitable for detecting effort in this population.

Performance on this task could also be influenced by iatrogenic factors, such as expectation of symptoms after injury or diagnosis, leading to differences in effort. However, participants did not know whether they were in the group with or without PCS, and without such categorization participants are less likely to be influenced by iatrogenic factors in relation to PCS. Participants could be influenced by expectation of symptoms after mTBI, but both mTBI groups would be equally influenced. Therefore, if there is an effect of poor effort in this study which is not detected by the 0-Back, then it is likely to be small, and unlikely to be the sole cause of the large deficit observed in cognitive performance.

The cognitive deficit seen in those participants with persistent PCS after mTBI may be due to a variety of underlying changes after injury. One putative mechanism which has begun to be explored is a disruption in connectivity in the default mode network (DMN; Mayer et al., 2011, 2012; Bonnelle et al., 2012; Sandrone, 2012; Sandrone and Bacigaluppi, 2012), which will need to be explored further in this participant grouping.

### Effect of PCS and co-variables on cognition

The hypothesis of this study was that those participants who report persistent PCS after mTBI would have greater cognitive deficit than participants who report no long-term symptoms after mTBI. Therefore, the data was investigated to see whether increased PCS symptoms would correlate with worse cognitive performance. In addition, as PCS symptom report is influenced by other factors, such as depression, anxiety, fatigue, and post-traumatic stress, it was considered important to explore whether these co-variables correlated with cognitive performance.

#### PCS symptoms

There was no significant correlation between performance and PCS symptom report for either task. However, there was a medium effect size association (but one which fell short of the significance threshold) between greater PCS symptom report and poorer n-Back performance in mTBI participants (Rho = 0.43, *p* = 0.004, Table 2), with no comparable association in control participants. There was also a medium effect size association for PVSAT performance and PCS symptoms in mTBI participants (Rho = 0.35, *p* = 0.02). Although these findings do not lend definitive support for a link between PCS symptoms and cognitive performance, the overall pattern of the results suggests PCS symptoms in mTBI participants may have stronger link to cognitive performance compared to control participants. This supports the hypothesis that the mechanisms leading to PCS after mTBI are distinct from those contributing to the PCS symptoms seen in the general population.

When reporting PCS symptoms using the RPQ, participants with mTBI are attributing the symptoms to the injury, whereas control subjects are not asked to make a specific attribution (Dean et al., 2012). It is therefore possible that an attribution bias is influencing the results, with a greater level of concern over the chronic cognitive effects of the injury causing participants with mTBI and persistent PCS to perform worse on the tasks. An attribution bias of this sort is likely to influence performance for all the cognitive tasks, as well as report of everyday cognitive failures (CFQ score). However, participants performance equally well in the 0-Back task, and CFQ score is equivalent in mTBI + PCS and control + PCS groups. An attribution bias may still be influencing the results to some extent, but not enough to explain the substantial differences seen in the working memory and information processing tasks whilst the sustained attention task (0-Back) is performed almost faultlessly. The influence of an attribution bias may be investigated further in follow-up studies being analyzed which use functional neuroimaging to look at underlying neural activity during this task.

#### Sleep quality

Night-time sleep quality (PSQI) was significantly worse in mTBI + PCS participants compared to both mTBI − PCS and Control − PCS, despite all groups having scores above or close to the threshold indicating a poor sleeper (Buysse et al., 1989). Sleep propensity (KSS) and sleepiness during the day (ESS) did not differ between the groups. It seems that although mTBI + PCS participants have poorer sleep, they do not report feeling sleepier during the daytime.

However, there was a correlation between poor PVSAT performance and poor sleep quality in mTBI participants (Rho = 0.62, *p* < 0.001, Table 2), with no comparable association in controls. This indicates that the poor sleep quality of some mTBI participants may be having an effect on aspects of their daytime functioning, even if there is no difference in reported daytime sleepiness and sleep propensity. Anecdotal evidence suggests that participants may revert to responding to all stimuli as non-targets when they felt under time pressure. This may also help to explain why there was no significant correlation between n-Back performance and sleep quality in mTBI participants (Rho = 0.31, *p* = 0.05).

Previous studies have investigated the role of sleep in the short and long-term after mTBI (Ayalon et al., 2007; Schreiber et al., 2008; Chaput et al., 2009), and the present study confirms the association between poor sleep and long-term outcome from mTBI. Sleep could be a risk factor for poor outcome from mTBI, with poor sleep prior to injury undermining subsequent recovery from PCS symptoms. Alternatively, the mTBI itself could trigger sleep problems in previously good sleepers, which in turn may prevent full recovery. In both cases sleep management programs provided after the initial injury could be a relatively simple treatment option to reduce long-term consequences of mTBI.

#### Post-traumatic stress disorder

PTSD (Bryant et al., 2009) is elevated in mTBI participants with PCS in comparison to mTBI participants without PCS. It is not possible to calculate PTSD in non-injured control participants. Therefore, we are unable to rule out the effect of PTSD on cognition, especially as the correlation between higher IES-R score and worse performance on the n-Back showed a medium effect size association (falling short of statistical significance; Rho = 0.43, *p* = 0.004, Table 2). Previous studies have used a control group that have sustained an injury to another part of the body without concurrent head injury (Bryant et al., 2009; Vanderploeg et al., 2009; Brenner et al., 2010a). Future studies will require a similar control group to investigate the effect of PTSD on cognitive performance in this paradigm.

#### Depression and anxiety

Depression and anxiety have the potential to affect both PCS symptom report and cognitive performance (Suhr and Gunstad, 2002; Iverson, 2006; Moore et al., 2006). There was no significant correlation between cognitive performance and depression in either experimental group. This is despite previous research suggesting a strong association between depression, PCS symptoms, and cognitive functioning (Suhr and Gunstad, 2002; Iverson, 2006; Sheline et al., 2010). The lack of any such effect here could be due to a difference in the sample tested (the majority studies recruit from hospitals, whereas this study recruited from a non-hosptial sample) or the depression scale used [this study used the HADS (Bjelland et al., 2002), whereas the Beck depression inventory (Beck et al., 1961) is sometimes used elsewhere]. However, there was a medium effect size association (falling short of statistical significance) between increased anxiety and worse PVSAT performance in mTBI participants (Rho = 0.44, *p* = 0.004). High anxiety in participants with mTBI could be related to the symptom of hypochondriacal concern as detailed in the ICD-10 criteria for PCS (WHO, 1992) [but not DSM-IV criteria (APA, 1994)]. Another possibility is that those with high anxiety may also have lower sleep quality, and it is this combination that is affecting PVSAT performance. This is an intriguing possibility, especially as there is a significant correlation between sleep quality and performance on the same task (PVSAT). Furthermore, participants in the mTBI + PCS and mTBI − PCS groups exhibited statistically different sleep quality, but no difference in anxiety levels. This requires further research, although the cognitive deficits seen in the mTBI + PCS group cannot be explained purely by increased anxiety levels as Control + PCS participants reported similar levels. Overall, it seems that the influence of anxiety on cognitive performance in this sample may be slight, and there is no tangible evidence of the influence of depression.

## Conclusion

This study investigated the long-term (>1 year) effects of mTBI on cognition, taking into account PCS in mTBI participants and PCS-like symptoms in control participants. Working memory and information processing speed was significantly impaired in mTBI participants with persistent PCS compared to mTBI participants without PCS and all non-head injured participants. Correlations between cognitive performance and symptoms were only observed for mTBI participants, with worse performance correlating with lower sleep quality, in addition to medium effect size associations (falling short of statistical significance) with higher PCS symptoms, PTSD, and anxiety.

The use of a control group with similar post-concussion symptoms to the participants with mTBI and PCS enabled us to distinguish to a certain extent the influence of confounders such as general cognitive failures, depression, anxiety, sleep quality, and sleepiness from the effect of the brain injury. These results suggest that the reduction in cognitive performance is not due to greater symptom report itself, but is associated to some extent with the initial injury. Furthermore, the results validate the utility of our participant grouping, and demonstrate its potential to reduce the variability observed in previous studies. However, the influence of sleep quality, and to a certain extent PTSD and anxiety, on cognitive performance requires further investigation. A longitudinal study using this protocol would be useful to elucidate the changes over time in these groups. Furthermore, some of the limitations inherent to meta-analyses of cognitive symptoms after mTBI (Pertab et al., 2009; Iverson, 2010; Rohling et al., 2009) may be alleviated using these participant groupings.

### Conflict of interest statement

The authors declare that the research was conducted in the absence of any commercial or financial relationships that could be construed as a potential conflict of interest.
